# Subcutaneous Chest Abscess Caused by Candida albicans Infection Following Laparoscopic Cholecystectomy in an Immunocompetent Patient: A Case Report

**DOI:** 10.7759/cureus.24573

**Published:** 2022-04-28

**Authors:** Hanako Yoshihara, Ibuki Kurihara, Hiroshi Hori, Takahiko Fukuchi, Hitoshi Sugawara

**Affiliations:** 1 Division of General Medicine, Department of Comprehensive Medicine 1, Saitama Medical Center, Jichi Medical University, Saitama, JPN; 2 Department of Internal Medicine, Minamiuonuma City Hospital, Niigata, JPN

**Keywords:** telogen effluvium, fluconazole, immunocompetent, subcutaneous abscess, candida albicans

## Abstract

Cases of subcutaneous abscess due to *Candida albicans *(*C. albicans*) infection are rare, even among immunocompromised patients. To our knowledge, there have only been eleven reports of such cases in adults, all of which presented with comorbidities of immunodeficiency, prior antibiotic administration, or skin breakdown following traumatic episodes or iatrogenic procedures.

We report a rare case of a 42-year-old Japanese woman with a subcutaneous abscess due to *C. albicans* infection. The patient was referred to our hospital with a chief complaint of gradually worsening lower left-sided chest pain. Nine months before admission, she underwent laparoscopic cholecystectomy (Lap-C) for acute cholecystitis at another hospital. She developed fever and was treated with cefotiam for three days followed by cefoperazone/sulbactam for four days.

One week after Lap-C, she began to feel pain in the lower left side of her chest. The chest pain worsened gradually and the fever persisted until two months before admission.

On admission, enhanced chest computed tomography revealed a left chest subcutaneous abscess located between the seventh and ninth rib. She underwent surgical percutaneous drainage, and the abscess cavity was cleaned. The pus culture revealed *C. albicans*, but the blood cultures were negative. We administered intravenous micafungin (150 mg daily) for 10 days, followed by oral fluconazole (600 mg daily). She experienced telogen effluvium during the period of fluconazole treatment but recovered after the cessation of fluconazole.

We also present a short review of the literature relating to subcutaneous candidal abscesses in patients over 15 years old.

## Introduction

*Candida*, which is opportunistic pathogenic yeast, is a very common fungus in the mouth and the gut that can cause systemic invasive fungal infections in humans, especially in immunocompromised patients. However, the subcutaneous candidal abscess is very rare, even in immunocompromised patients [1]. Management of such abscesses involves the administration of effective antifungal therapy and targeted source control [2].

We present the case report of an immunocompetent patient with a subcutaneous abscess in the lower left chest caused by *Candida albicans* (*C. albicans*) infection, which developed after laparoscopic cholecystectomy (Lap-C). We also present a short review of the literature relating to subcutaneous candidal abscesses in patients over 15 years old.

## Case presentation

**Figure 1:**
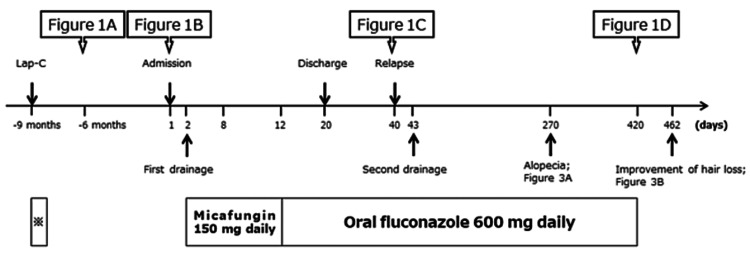
Clinical course of the progress of imaging studies, drainage, hair depletion, and antifungal medications. The asterisk (*) indicates the medications, including cefotiam for three days followed by cefoperazone/sulbactam for four days. Lap-C, laparoscopic cholecystectomy.

A 42-year-old Japanese woman with Hashimoto’s disease was referred to our hospital with the chief complaint of gradually worsening sharp pain in the lower left side of the chest on exertion. Figure 1 illustrates the clinical course of the patient. The patient was receiving oral levothyroxine (50 µg) for her condition and underwent Lap-C for acute cholecystitis at another hospital nine months prior to presentation at our department. After surgery, she developed a fever and was treated with cefotiam for three days followed by cefoperazone/sulbactam for four days. About one week after Lap-C, she began to feel pain in the lower left side of her chest but was discharged without additional treatment.

**Figure 2:**
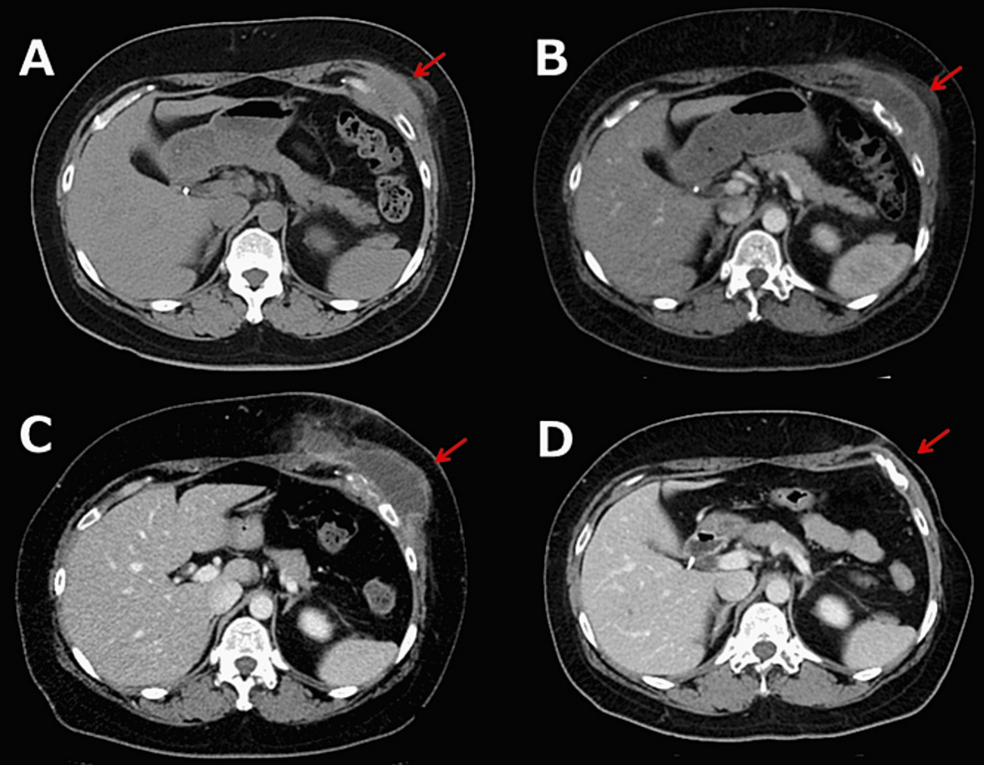
Whole-body computed tomography (CT) shows the course of the left subcutaneous abscess. (A) A whole-body CT at the previous hospital where the Lap-C was performed. It revealed a fluid density area around the lower left rib in the anterior chest wall, which was suspected to be a “hematoma”. (B) A contrast-enhanced whole-body CT on admission revealed a fluid density area around the lower left side of the ribs. (C) The subcutaneous abscess grew again 20 days after discharge. (D) The subcutaneous had completely resolved. Red solid arrows indicated the subcutaneous abscess.

Three months after the Lap-C (six months prior to admission), whole-body computed tomography (CT) was performed at the hospital where the Lap-C was performed. This revealed fluid density around her lower left rib in the anterior chest wall, which was suspected to be a “hematoma” (Figure 2A). The chest pain was treated with nonsteroidal anti-inflammatory drugs. However, the pain in the lower left side of her chest gradually worsened and the fever persisted until she was referred to our hospital.

Physical examination revealed tenderness and swelling in the lower left side of her chest, and the patient was admitted. Contrast-enhanced whole-body CT scan on admission revealed that the fluid density area around the lower left side of her ribs had grown in size and measured 6.5 cm in diameter (Figure 2B). Laboratory tests revealed a white blood cell count of 10.24 × 10^6^/L, hemoglobin of 11.9 g/dL, platelet count of 347 × 10^3^/µL, aspartate aminotransferase of 12 U/L, alanine aminotransferase of 18 U/L, lactate dehydrogenase of 137 U/L, creatinine of 0.54 mg/dL, HbA1c of 5.9%, and C‐reactive protein of 2.31 mg/dL (Table 1).

**Table 1:** Laboratory test results on admission HbA1c, Hemoglobin A1c; AST, Aspartate aminotransferase; ALT, Alanine aminotransferase; LDH, Lactate dehydrogenase; CK, Creatine phosphokinase, ALP, Alkaline phosphatase; ɤ-GTP, Gamma-glutamyl transpeptidase; CRP, C‐reactive protein; BUN, Blood urea nitrogen; IgG, Immunoglobulin G; IgA, Immunoglobulin A; IgM, Immunoglobulin M; HIV, Human immuno-deficiency virus

Tests	Data	Reference range	
Complete blood count			
White blood cells	10.24× 10^6^	3.9–9.3 × 10^6^/L	
Neutrophils	59	40%–70%	
Lymphocytes	36	22%–44%	
Monocytes	4	4%–11%	
Atypical lymphocytes	1	0%–2%	
Red blood cells	4.36×10^9^	3.5–5.0 × 10^9^/L	
Hemoglobin	11.9	≥12g/dL	
Platelets	374×10^3^	150–450 × 10^3^/µL	
Chemistry			
HbA1c	5.9	4.0%–5.6%	
Blood glucose (fasting)	95	70-109 mg/dL	
Total protein	7.5	6.6–8.1 g/dL	
Albumin	3.4	4.1–5.1 g/dL	
Total bilirubin	0.23	0.4–1.5 mg/dL	
AST	12	13–30 U/L	
ALT	18	7–23 U/L	
LDH	137	124–222 U/L	
CK	42	41–153 U/L	
ALP	219	106–322 U/L	
ɤ-GTP	51	9–23 U/L	
CRP	2.31	0–0.14 mg/dL	
Sodium	139	138–145 mmol/dL	
Potassium	4.5	3.6–4.8 mmol/L	
Chloride	105	100–110 mmol/L	
Calcium	10.2	8.4–10.1 mg/dL	
Phosphorus	4.0	2.7–4.6 mg/dL	
Magnesium	2.0	1.7–2.5 mg/dL	
BUN	8	8–20 mg/dL	
Creatinine	0.54	0.46–0.79 mg/dL	
IgG	1689	870–1700 mg/dL	
IgA	301	110–410 mg/dL	
IgM	77	46–260 mg/dL	
HIV	Negative	Nagative	

**Figure 3:**
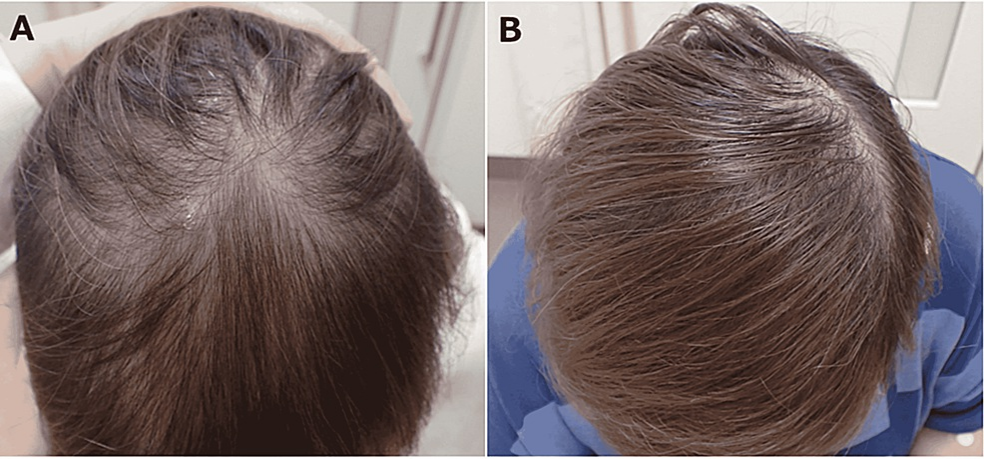
(A) Telogen effluvium during treatment with fluconazole 600 mg daily, orally for nine months. (B) The patient’s hair regrew over six weeks after the cessation of fluconazole.

On the second day of admission to our hospital, the patient underwent percutaneous surgical drainage, and the abscess cavity was drained and cleaned. A sample of the drained fluid was sent for culture, which was found to be positive for *C. albicans*. Blood cultures were negative. She was treated intravenously with micafungin 150 mg daily for 10 days followed by oral fluconazole 600 mg daily. She was discharged from the hospital on day 20 in good physical condition. Although she continued to take the same dose of fluconazole, the subcutaneous abscess began to grow again 20 days after discharge (Figure 2C). She underwent further surgical drainage and continued the same dose of fluconazole for nine months, during which her hair started to fall out (Figure 3A). Although we considered that fluconazole was responsible for her telogen effluvium, we continued this agent for another five months until the abscess had completely resolved (Figure 2D) because it was the only oral medication covered by the National Health Insurance in Japan that she was able to afford. Her hair regrew over six weeks after ceasing fluconazole (Figure 3B) and she had no relapse of the subcutaneous abscess after the cessation.

## Discussion

This report describes an immunocompetent patient with a subcutaneous abscess in the lower left side of the chest, caused by *C. albicans* which developed one month after Lap-C. The clinical course of this patient raised two important clinical issues: (1) Subcutaneous candidal abscesses can occur even in immunocompetent patients with skin breakdown and prior antibiotics administration and (2) long-term fluconazole administration can cause telogen effluvium.

To the best of our knowledge, there have only been 10 other reports of *Candida* *species* subcutaneous abscesses in patients >15 years old from 1963 to March 2022 [1,3-11]. These case reports are summarized in Table 2. Of the 11 patients reported in these publications, nine had abscesses due to *C. albicans* and five patients had diabetes. All these patients with a subcutaneous candidal abscess had a background history of immunodeficiency, prior antibiotic administration, or skin breakdowns, such as traumatic episodes or iatrogenic procedures.

**Table 2:** Summary of the reported cases of Candida subcutaneous abscesses in adults

Patient	Age	Sex	Comorbidities	Skin breakdown before onset	Candida spices	Location	Pre-antibiotics	Reference
1	17	F	Diabetes	Daily insulin injection	C. albicans	Upper thighs	No	(3)
2	32	M	Tuberculosis bowel perforation	NA	C. albicans	Left lower thoracic	Yes	(4)
3	36	M	HIV (CD4+ lymphocyte count 61/µL) Liver cirrhosis due to HBV infection	Intravenous drug user	C. albicans	Right upper thoracic wall	No	(5)
4	49	F	Diabetes	A history of dipping of sunflower stick on her foot/daily insulin injection	C.glabrata	Left foot	No	(6)
5	50	M	Cushing’s syndrome (long-term corticosteroid) uncontrolled diabetes Diabetes	Daily insulin injection	C. albicans	Both legs	No	(1)
6	57	F	Undiagnosed diabetes	A history of self-administering acupuncture at home using a nondisposable needle without an adequate skin disinfection	C. albicans	Left periorbital area	Yes	(7)
7	59	M	Bedridden for the past 2 months because of subarachnoid hemorrhage Long-term corticosteroid	Intravenous catheter (into the left great saphenous vein at the medial malleolus)	C. albicans	Left knee	Yes	(8)
8	59	F	A buccal-space infection Diabetes	the extraction of left upper second premolar and first molar teeth	C. albicans	left cheek	Yes	(9)
9	68	M	Acute myelocytic leukemia Neutropenic fevers (due to cytarabine and daunorubicin)	Injections of heparin to the abdominal wall	C.krusei	Left side of the abdomen	Yes	(10)
10	86	F	Steroid user (due to sciatica)	Rectal bleeding	C. albicans	Perirectal abscess	Yes	(11)
11	42	F	Hashimoto’s disease	Laparoscopic cholecystectomy 9 months prior to admission	C. albicans	left lower thoracic	Yes	Present

Our patient is a very rare case of subcutaneous candidal abscess in a 42-year-old female immunocompetent patient after Lap-C. The incidence of infection at the surgical site of Lap-C is significantly lower than that of open cholecystectomy [12], and in our patient, the location of the surgical wound did not correspond to the subcutaneous candidal abscess. We believe that the candidal abscess was not caused by surgical site infection, but possibly by microbial substitution due to the use of antibiotics to manage the fever, the patient had following Lap-C, one month prior to onset.

In the present patient, telogen effluvium was also observed due to long-term administration of fluconazole. Alopecia associated with fluconazole therapy (telogen effluvium) developed three months after initiation of this drug, the incidence reportedly being 12.5%-20.0% in patients taking 400 mg/day for two months or longer [13].

In Japan, long-term administration of fluconazole is often used to prevent or treat deep fungal infections associated with chemotherapy-related neutropenia but rarely at other times. Therefore, it is expected that the appearance of fluconazole-induced alopecia will not be noticed in patients in whom hair loss has already occurred due to systemic chemotherapy.

While this adverse effect was distressing in the present case, it resolved when fluconazole therapy was discontinued.

## Conclusions

Subcutaneous candidal abscesses can occur even in immunocompetent patients with skin breakdown and prior antibiotics administration. They are rare but require long-term fluconazole administration. Hence, it is pertinent to consider the adverse effect of reversible alopecia.
